# Post-cholecystectomy Clip Migration: A Case Report

**DOI:** 10.7759/cureus.58580

**Published:** 2024-04-19

**Authors:** Renisha Singh, Praveen Arumugam, Kushagra Mathur, Abhishek Deo

**Affiliations:** 1 Internal Medicine, Max Super Speciality Hospital, New Delhi, IND; 2 Gastroenterology and Hepatology, Max Super Speciality Hospital, New Delhi, IND

**Keywords:** endoclipping, post-ercp, endoscopic clip, laparoscopic cholecystectomy. clip migration. hem-o-lok clip. common bile duct (cbd). endoscopic retrograde cholangiopancreatography (ercp). magnetic resonance cholangiopancreatography (mrcp), post operative complication

## Abstract

Surgical clip migration into the common bile duct (CBD) with subsequent stone formation is an exceedingly rare complication following both laparoscopic and open cholecystectomy, with fewer than 100 cases reported in the literature. Herein, we present the case of a 78-year-old female who presented with abdominal pain and dark urine six years after an open cholecystectomy. Her abdominal ultrasonography revealed no abnormalities, with only mild derangements noted in liver function tests. However, computed tomography of the abdomen unveiled a single metallic surgical clip lodged within the CBD, surrounded by a bile stone, alongside another clip at the gallbladder fossa. The patient underwent endoscopic retrograde cholangiopancreatography (ERCP), during which the clip was successfully removed. The procedure has utilized SpyGlass cholangioscopy. While clip migration into the CBD remains a rare phenomenon, it should be considered in the differential diagnosis of patients presenting with obstructive jaundice or biliary colic post-cholecystectomy. Minimally invasive management by ERCP is the procedure of choice for migrated clips-related complications but surgical common bile duct exploration may be necessary. This case highlights the importance of vigilance and prompt intervention in managing post-cholecystectomy clip migration (PCCM) but potentially serious postoperative complications.

## Introduction

Cholecystectomy stands as the cornerstone in the management of gallstone disease, offering definitive treatment for symptomatic gallstones [1]. While laparoscopic cholecystectomy has become the preferred approach due to its minimally invasive nature and associated benefits, including reduced postoperative pain and shorter hospital stays, it is imperative to recognize that complications can still arise regardless of the surgical technique employed. Common complications following cholecystectomy, whether laparoscopic or open, encompass bile duct injury, leakage, infection, strictures, and post-cholecystectomy clip migration (PCCM) [2].

Post-cholecystectomy clip migration represents a rare yet noteworthy complication, occurring when surgical clips utilized to seal the cystic duct migrate into the common bile duct (CBD). Although infrequent, this complication warrants attention due to its potential to lead to obstructive jaundice, biliary colic, or even more serious consequences if left untreated [3-5]. Existing literature suggests that the majority of PCCM cases can be successfully managed through endoscopic retrograde cholangiopancreatography (ERCP), a minimally invasive procedure aimed at removing the migrated clip and resolving associated biliary obstruction. However, approximately 20% of cases may necessitate alternative therapeutic approaches beyond ERCP, underscoring the importance of recognizing and promptly addressing this complication to optimize patient outcomes [3,4]. As such, this case report aims to contribute to the existing body of knowledge surrounding PCCM by presenting a clinical case of clip migration into the CBD following cholecystectomy.

## Case presentation

A 78-year-old female presented with several weeks of epigastric pain, nausea, and a recent onset of dark urine. Her past surgical history revealed an open cholecystectomy performed six years ago. Physical examination revealed a febrile status, with a soft abdomen demonstrating slight epigastric tenderness and a negative Murphy's sign. Laboratory investigations unveiled markedly deranged liver function tests (LFTs), suggestive of a cholestatic pattern. Ultrasonography (USG) of the entire abdomen yielded unremarkable findings, prompting further evaluation with a computed tomography (CT) scan of the abdomen.

**Figure 1:**
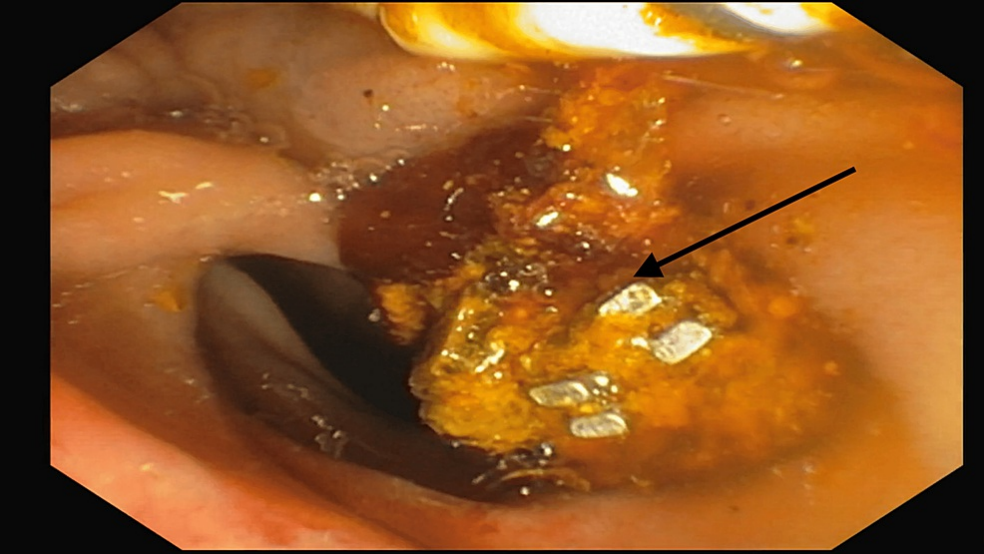
The image of clips (black arrow) obtained during ERCP demonstrated the presence of a metallic clip in the centre of a common bile duct stone. The "stone clip" combination was successfully removed. ERCP: Endoscopic retrograde cholangiopancreatography

The CT scan revealed a post-cholecystectomy status with a metallic clip present in the gallbladder fossa, alongside biliary dilatation and an elongated metallic density in the mid-common bile duct (CBD), suggestive of clip displacement from the gallbladder fossa. Subsequently, endoscopic retrograde cholangiopancreatography (ERCP) was performed, during which the metallic clip (Figure 1) was successfully removed in a single attempt, along with a stone from the CBD. The patient was closely monitored and treated post-procedure while inpatient and was discharged following normalization of LFTs.

This case highlights a rare presentation of post-cholecystectomy complications, emphasizing the importance of considering such possibilities in patients presenting with abdominal symptoms after cholecystectomy. Additionally, it underscores the role of ERCP as an effective therapeutic modality in managing complications related to surgical clip migration into the CBD.

## Discussion

Post-cholecystectomy clip migration (PCCM) represents a rare but recognized complication, with approximately 100 cases reported in the literature to date. Most cases manifest months following cholecystectomy, typically presenting with symptoms resembling biliary colic. Diagnosis is often facilitated through abdominal ultrasound, with successful management achieved via endoscopic retrograde cholangiopancreatography (ERCP) in the majority of cases. However, a small subset necessitates surgical exploration of the bile duct or percutaneous transhepatic drainage [3-6].

Several hypotheses have been proposed to elucidate the pathogenesis of PCCM. Kitamura et al. [7] postulated that pressure exerted by the liver may cause inversion of the cystic duct stump into the common bile duct (CBD), leading to subsequent necrosis and migration of surgical clips. Another theory implicates incorrect clip placement, resulting in bile duct leakage and subsequent inflammation, leading to erosion of the cystic stump and CBD wall, and facilitating clip migration and stone formation.

Additionally, it has been suggested that incorrect surgical clip placement may result in bile duct leakage and biloma formation. Subsequent inflammation surrounding the area can lead to erosion of the cystic stump and CBD wall, allowing surgical clips to migrate into the CBD. The clips then act as a nidus for bile aggregation, leading to stone formation and bile duct obstruction [4,8].

While fatalities directly attributable to PCCM are not documented, severe complications such as cholangitis and pancreatitis can ensue [3,8-10]. To mitigate these risks, meticulous attention must be paid to clip placement during surgery, with limitations placed on the number of clips used [3,4]. Alternatives such as absorbable sutures have been proposed but carry the risk of gallstone formation [11,12].

In our case, lithotripsy with SpyGlass cholangioscopy emerged as a potentially safer, non-invasive therapeutic option for refractory bile duct stones [13,14]. SpyGlass cholangioscopy allows for direct visualization of the bile duct, facilitating accurate diagnosis and targeted treatment [13,15]. However, challenges related to equipment availability and training may limit its widespread adoption.

In conclusion, PCCM poses a rare but significant complication following cholecystectomy, necessitating heightened vigilance during surgical procedures and consideration of alternative therapeutic modalities in refractory cases. Further research is warranted to refine management strategies and optimize patient outcomes in this challenging clinical scenario.

## Conclusions

In conclusion, post-cholecystectomy clip migration (PCCM) should be included in the differential diagnosis for patients presenting with obstructive jaundice symptoms following cholecystectomy, regardless of the time elapsed since the procedure. This case underscores the importance of maintaining a high index of suspicion for rare but potentially serious complications in postoperative patients. Furthermore, our case highlights the utility of employing endoscopic retrograde cholangiopancreatography (ERCP) with SpyGlass cholangioscopy and lithotripsy for the extraction of challenging bile duct stones associated with PCCM. These innovative technologies offer promising solutions for the management of complex cases, allowing for precise visualization and targeted intervention.
